# The Use of the Calcitonin Minimal Recognition Module for the Design of DOPA-Containing Fibrillar Assemblies

**DOI:** 10.3390/nano4030726

**Published:** 2014-08-20

**Authors:** Galit Fichman, Tom Guterman, Lihi Adler-Abramovich, Ehud Gazit

**Affiliations:** 1 Department of Molecular Microbiology and Biotechnology, George S. Wise Faculty of Life Sciences, Tel Aviv University, Tel Aviv 6997801, Israel; E-Mails: galitpe1@post.tau.ac.il (G.F.); tomguter@mail.tau.ac.il (T.G.); lihia@tauex.tau.ac.il (L.A.-A.); 2 Department of Materials Science and Engineering, Iby and Aladar Fleischman Faculty of Engineering, Tel Aviv University, Tel Aviv 6997801, Israel

**Keywords:** self-assembly, amyloid, calcitonin, DOPA, L-3,4-dihydroxyphenylalanine

## Abstract

Amyloid deposits are insoluble fibrous protein aggregates, identified in numerous diseases, which self-assemble through molecular recognition. This process is facilitated by short amino acid sequences, identified as minimal modules. Peptides corresponding to these motifs can be used for the formation of amyloid-like fibrillar assemblies *in vitro*. Such assemblies hold broad appeal in nanobiotechnology due to their ordered structure and to their ability to be functionalized. The catechol functional group, present in the non-coded L-3,4-dihydroxyphenylalanine (DOPA) amino acid, can take part in diverse chemical interactions. Moreover, DOPA-incorporated polymers have demonstrated adhesive properties and redox activity. In this work, amyloid-like fibrillar assemblies were formed through the self-assembly of a pentapeptide containing DOPA residues, Asp-DOPA-Asn-Lys-DOPA. The design of this peptide was based on the minimal amyloidogenic recognition motif of the human calcitonin hormone, Asp-Phe-Asn-Lys-Phe, the first amyloidogenic pentapeptide identified. By substituting phenylalanine with DOPA, we obtained DOPA-functionalized amyloid-like assemblies in water. Electron microscopy revealed elongated, linear fibril-like nanometric assemblies. Secondary structure analysis indicated the presence of amyloid-characteristic β-sheet structures as well as random coil structures. Deposition of silver on the DOPA-incorporated assemblies suggested redox activity and demonstrated the applicative potential of this novel nanobiomaterial.

## 1. Introduction

Molecular self-assembly is a prevalent phenomenon in biological systems. Non-covalent interactions lead to the formation of complex supramolecular structures at the nano-scale from simple building blocks, including proteins and peptides [1,2]. A pathologically significant facet of these molecular recognition and self-assembly processes is the formation of amyloid fibrils. These are proteinaceous insoluble deposits that were identified in numerous human disorders including Alzheimer’s and Parkinson’s diseases [3,4]. When amyloids are being formed, soluble proteins or polypeptides self-assemble into insoluble ordered fibrils, a process coupled with a secondary structure transition to a predominantly β-sheet conformation [5]. In most amyloid fibrils, the polypeptide backbone of the β-strands is perpendicular to the fibril axis, a configuration known as cross-β structure [6,7].

Amyloid fibrils self-assemble through molecular recognition facilitated by short amino acid sequences found in amyloidogenic proteins or polypeptides, which were identified as minimal amyloidogenic recognition modules [8]. These modules can serve as initiators and facilitators of aggregation and vary between amyloidogenic proteins and polypeptides. By employing a reductionist approach, *in vitro* studies utilizing short synthetic peptides as model systems led to the discovery of minimal recognition modules in numerous amyloidogenic proteins and polypeptides [9,10,11,12,13]. One such module was identified in human calcitonin (hCT), a 32-residue polypeptide hormone which plays a role in calcium-phosphate homeostasis [14]. hCT can form amyloid fibrils *in vivo* and the fibrils were implicated in the pathogenesis of medullary thyroid carcinoma [15]. *In vitro* amyloid fibril formation by synthetic hCT was reported as well [16]. The *in vitro* formation of hCT amyloid fibrils is affected by the pH of the medium and fibrils are formed mainly in neutral aqueous solution [17,18]. Our group has previously identified the sequence Asp-Phe-Asn-Lys-Phe as the minimal amyloidogenic recognition module of hCT [19]. This pentapeptide, spanning residues 15–19 of hCT, forms amyloid fibrils *in vitro* at neutral pH in aqueous solutions with remarkable similarity to the fibrils formed by the full-length hCT. The hCT minimal recognition module was the first pentapeptide to be identified as forming canonical amyloids [8]. Due to its short length, this pentapeptide was studied by experimental as well as by computational methods [20,21,22,23].

**Figure 1:**
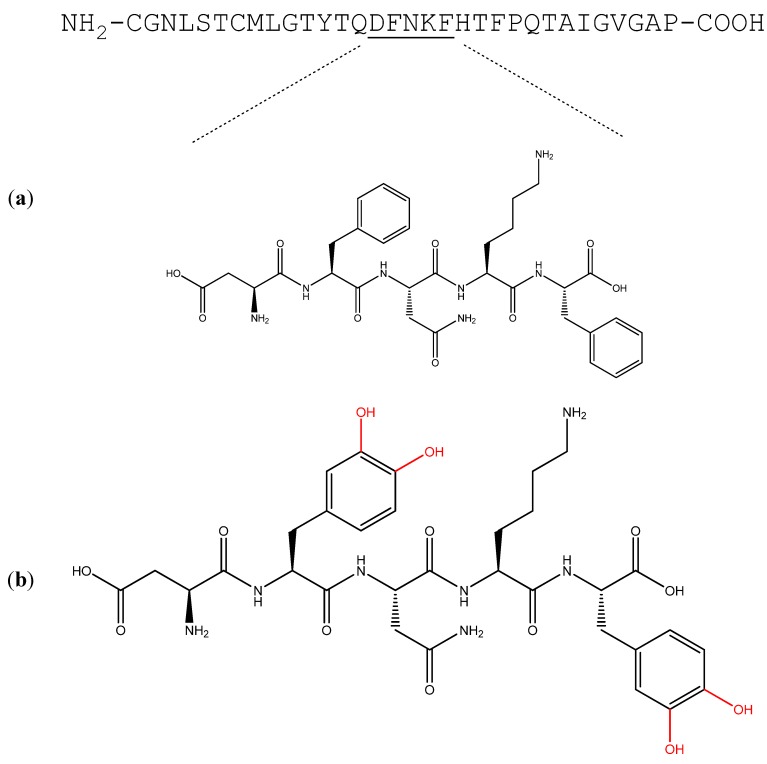
A L-3,4-dihydroxyphenylalanine (DOPA)-containing building block inspired by the human calcitonin (hCT) recognition module. (**a**) Amino acid sequence of hCT. Underlined are residues 15–19, which constitute the minimal amyloidogenic recognition module of hCT; the chemical structure of the module appears below; (**b**) the chemical structure of the hCT-inspired DOPA-containing pentapeptide, Asp-DOPA-Asn-Lys-DOPA. The catechol hydroxyl substituents appear in red.

Self-assembly of amyloid-like structures has been a subject of much interest in nanobiotechnology. Due to their ability to self-assemble into ordered nanostructures that may also be chemically and biologically functionalized, amyloidogenic peptides are regarded as promising building blocks for various nanobiotechnological applications [24,25,26,27,28,29,30,31,32]. Pertinent to this is functionalization by the catechol group. This functional group can take part in diverse chemical interactions including hydrogen bonds, metal-ligand complexation, Michael-type addition, π–π interactions and quinhydrone-type charge-transfer complexation [33,34]. Adhesive properties were demonstrated by catechol-incorporated materials which were used as binding agents for both organic and inorganic surfaces [35,36]. The non-coded L-3,4-dihydroxyphenylalanine (DOPA) amino acid, containing the catechol group, has been incorporated into synthetic polymers which, by utilizing the redox activity of the catechol group, were used for the preparation of antifouling surfaces [37], metal nanoparticles [38] and antimicrobial films and hydrogels [39,40]. Recent studies have reported the self-assembly of DOPA-containing building blocks into nanometric fibers [41,42] and the unique functionality of such assemblies has been demonstrated [43]. Here, a short synthetic peptide, the minimal amyloidogenic recognition module of hCT, was designed to contain two DOPA moieties, substituting the phenylalanine residues, resulting in a unique building block, the Asp-DOPA-Asn-Lys-DOPA pentapeptide (Figure 1). This pentapeptide retained the ability to spontaneously self-assemble *in vitro* into amyloid-like fibrillar assemblies in water. The obtained assemblies displayed structural properties characteristic of amyloids as well as characteristics of DOPA-containing polypeptides. Functional assessment of the assemblies suggested redox activity and demonstrated the applicative potential of this novel nanobiomaterial.

## 2. Results and Discussion

### 2.1. Morphological Characterization of the Peptide Assemblies

**Figure 2:**
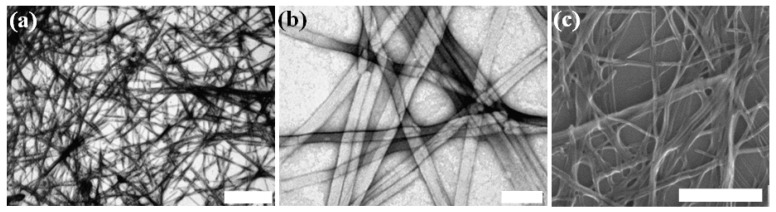
The DOPA-containing peptide Asp-DOPA-Asn-Lys-DOPA assemblies. High-resolution microscopy of fibrillar assemblies formed by 6 mM Asp-DOPA-Asn-Lys-DOPA in water. (**a**,**b**) Transmission electron microscopy (TEM) micrographs, negative staining was applied; Scale bars represent 2 µm and 100 nm; (**c**) scanning electron microscopy (SEM) micrograph, scale bar represents 1 µm.

Lyophilized Asp-DOPA-Asn-Lys-DOPA peptide was dissolved in water followed by the application of bath-sonication for 10 min. Concentrations of 100 µM to 15 mM were tested and a viscous turbid solution was obtained in all cases. We used transmission electron microscopy (TEM) to examine the samples, which revealed a network of fibrillar assemblies (Figure 2a,b). The observed fibrillar assemblies were mostly linear, unbranched and extending to the length of micrometers. The width of individual fibrillar assemblies varied from 15 to 85 nm and lateral bundling of the assemblies was observed. Such morphological features are common to amyloids and amyloid-like structures. In addition, scanning electron microscopy (SEM) was used to examine the three-dimensional nature of the Asp-DOPA-Asn-Lys-DOPA assemblies (Figure 2c). It should be noted that the fibrillar assemblies formed by the unmodified hCT minimal recognition module share the general morphology as observed, yet are considerably finer (Figure S1).

### 2.2. Secondary Structure Analysis of the Peptide Assemblies

**Figure 3:**
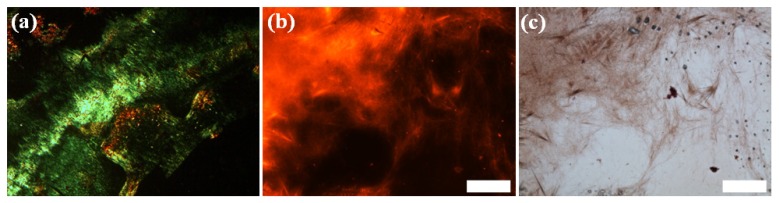
Congo Red (CR) staining of Asp-DOPA-Asn-Lys-DOPA. The peptide sample was stained with CR and examined by (**a**) polarized optical microscopy and by (**b**) fluorescence microscopy; (**c**) brightfield image corresponding to the fluorescence microscopy micrograph. Scale bars represent 100 µm.

A hallmark of the amyloid cross-β structure is apple-green birefringence of the dye Congo Red (CR) under polarized light when bound to amyloid fibrils [44]. This can be supported by CR fluorescence, which gives red-orange emission (616 nm) upon green excitation (510–560 nm) [45,46]. When Asp-DOPA-Asn-Lys-DOPA samples were dried and stained with CR, apple-green birefringence (Figure 3a) and red-orange fluorescence (Figure 3b) were observed while virtually none were observed in control samples of CR only or of the peptide without staining. Another dye which is extensively used for detecting amyloid fibrils is Thioflavin-T (ThT) [47,48]. Fresh peptide solutions were incubated with ThT for 3 h and imaged using confocal laser scanning microscopy (CLSM). Although ample fibrillar assemblies were observed, only weak fluorescence was detected (Figure S2). Similar results were obtained when fluorescence was measured using an automated plate reader (data not shown). Although amyloids usually bind ThT, reports of poor or no binding to ThT exist [49]. This is usually attributed to the lack of sequential β-strands or to an increased presence of the positively charged amino acid lysine in the amyloid, as could be in the case of Asp-DOPA-Asn-Lys-DOPA. Interestingly, the lack of ThT binding was also reported for the unmodified hCT minimal recognition module [50].

**Figure 4:**
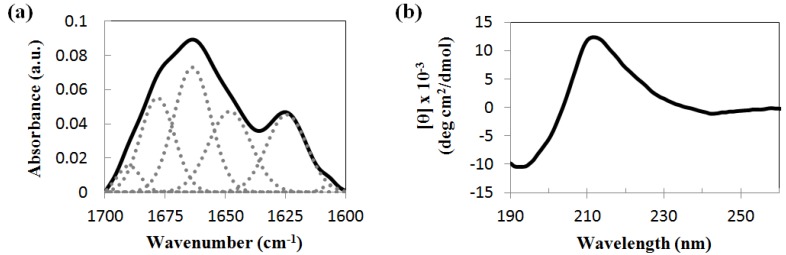
Secondary structure analysis of Asp-DOPA-Asn-Lys-DOPA. (**a**) Fourier transform infrared (FTIR) spectrum of dried 6 mM solution sample. The spectrum was analyzed by curve-fitting the second derivative of the amide I’ region; (**b**) circular dichroism (CD) spectrum of 3 mM solution at 25 °C.

Further analysis was performed using Fourier transform infrared (FTIR) spectroscopy. Asp-DOPA-Asn-Lys-DOPA was dissolved in deuterium oxide to a concentration of 6 mM, incubated overnight, dried and analyzed. The second derivative of the amide I’ region (1600–1700 cm^−1^) was curve-fitted and component bands were assigned to secondary structure elements [51,52,53] (Figure 4a). Distinct peaks were found at approximately 1625 and 1678 cm^−1^ and are attributed to the presence of β-sheet structures. A third peak, at approximately 1648 cm^−^^1^, is attributed to random coil conformations and a fourth peak at 1664 cm^−1^ can be attributed to either random coil or β-turn structures [52,54,55]. A fifth, minor component at 1690 cm^−1^ can be attributed to either β-turn or β-sheet structures and may suggest the presence of antiparallel β-sheet structures in conjunction with the 1625 cm^−1^ peak. Indeed, an antiparallel β-sheet structure, albeit with different peak positions, was previously reported for fibrils formed by the unmodified hCT minimal recognition module [19].

To examine the secondary structure of Asp-DOPA-Asn-Lys-DOPA in solution, freshly made 6 mM solutions were diluted to a final concentration of 3 mM and circular dichroism (CD) spectroscopy in the far-UV region was performed at 25 °C. Weak negative ellipticity was observed at approximately 235–250 nm, followed by a weak positive shoulder around 230 nm, a positive maximum at 209–212 nm and a negative maximum at 193 nm (Figure 4b). This spectral profile is ambiguous and can be interpreted in several ways. First, a similar CD spectrum has been previously reported for the highly-polymerized poly(Lys-Lys-Lys-DOPA) sequential polypeptide in water at 25 °C [56]. A negative maximum near 195 nm indicated that the sequential polypeptide adopted a random coil conformation under these conditions, whereas the positive ellipticity around 220 nm was attributed to transitions of the catechol side-chains and not to secondary structure elements; this therefore suggests that Asp-DOPA-Asn-Lys-DOPA adopts a random coil conformation in solution, as was also evident by component bands of the amide I’ region. However, this interpretation does not account for the β-sheet structures suggested by the FTIR and CR assays and cannot explain the amyloid-like morphology of the peptide assemblies. The presence of β-sheets may be inferred from a second type of CD spectra. These spectra are of short peptides that adopt a β-turn conformation, with characteristic positive ellipticity between approximately 200 and 235 nm and weak negative ellipticity between 235 and 250 nm in some of the cases [57,58]. Not only does the spectral profile of these peptides resemble the measured spectrum, it has also been reported that such peptides can self-assemble into supramolecular β-sheets and amyloid-like fibrils [59]. Interestingly, β-turn structures were reported for the unmodified hCT minimal recognition module as well [60], albeit its CD spectral profile differed markedly from the measured spectrum of Asp-DOPA-Asn-Lys-DOPA. Taken together, it is suggested that Asp-DOPA-Asn-Lys-DOPA adopts either a random coil conformation or a β-turn conformation in water and that the β-turn structures self-assemble into supramolecular β-sheets, giving rise to amyloid-like fibrillar assemblies.

**Figure 5:**
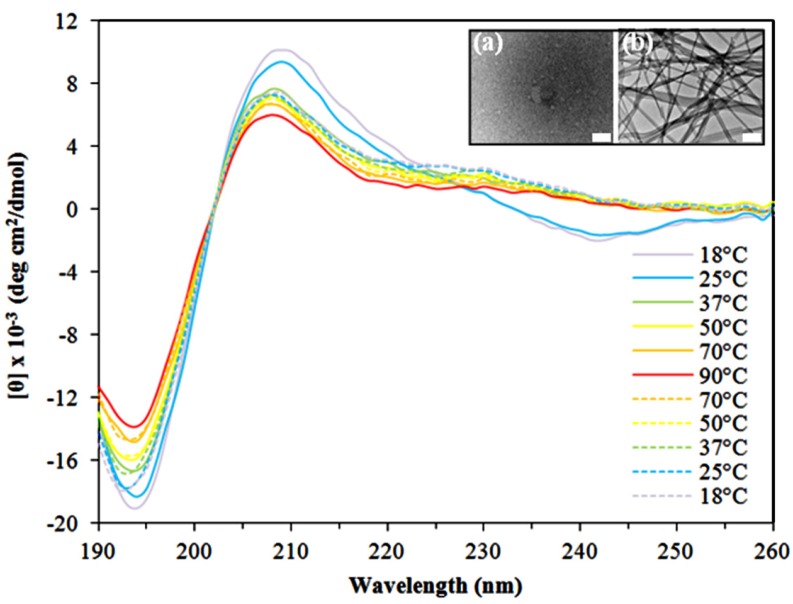
Temperature-dependent CD of 0.15 mM Asp-DOPA-Asn-Lys-DOPA in water. The temperature was increased in a stepwise fashion from 18 to 90 °C then similarly decreased to 18 °C. Insets show TEM micrographs of the CD cuvette content at (**a**) the end of the experiment and of (**b**) a control solution incubated at room temperature. Negative staining was not applied. Scale bars of the insets represent 200 nm.

To further elucidate the structural properties of Asp-DOPA-Asn-Lys-DOPA, temperature-dependent CD was performed. Freshly made 6 mM peptide solutions in water were diluted to a final concentration of 0.15 mM and CD spectra were collected during a stepwise increase in temperature from 18 to 90 °C and a subsequent stepwise decrease to 18 °C. Throughout this process, the spectral profile retained its distinct features (Figure 5a). However, intensity loss was observed as the temperature increased, with a significant loss occurring near the ~210 nm positive peak and in the 235–250 nm negative region. This effect seemed irreversible as only minor intensity gain was observed upon the subsequent temperature decrease and suggests a lower content of β-turn structures or possibly a deformation of the turn structure due to temperature elevation. The increase in temperature has also led to intensity loss in the 195 nm band, which regained its intensity during the subsequent temperature decrease; this reversible change can be explained in light of a temperature-dependent change in the backbone rigidity of random coil structures, which is linked to the effective length of the spectroscopic unit [61]. In a subsequent TEM examination of the heated and cooled solution, characteristic fibrillar assemblies were not observed (Figure 5, inset a). In contrast, a control solution which was incubated at room temperature contained the characteristic assemblies (Figure 5, inset b). The absence of assemblies following temperature elevation seems irreversible, at least during the experimental timeframe, as the characteristic assemblies were not detected by TEM even after approximately 24 h following the exposure to elevated temperatures (data not shown). The ensuing ultrastrcutural impairment seems to arise from a conformational change of the peptide since its chemical composition did not change following temperature elevation as evident by liquid chromatography–mass spectrometry (LC–MS) analysis (data not shown).

**Figure 6:**
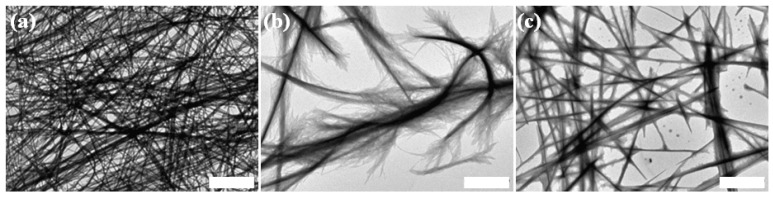
The ultrastructural effect of subjecting Asp-DOPA-Asn-Lys-DOPA assemblies to 37 °C. 6 mM peptide solution in water was allowed to assemble at room temperature for four days then sampled for TEM (**a**) after additional overnight incubation at room temperature or (**b**) after additional overnight incubation at 37 °C; (**c**) the same solution aliquot presented in the previous panel, sampled after 8 h recovery at room temperature. For all samples, negative staining was not applied. Scale bars represent 2 µm.

Since the first significant change in the CD signal was observed when the temperature was increased from 25 to 37 °C, we sought to examine the ultrastructural effect of subjecting the assemblies to this particular temperature. To this end, a peptide solution at a concentration of 6 mM was allowed to self-assemble at room temperature for four days then incubated overnight at 37 °C. TEM samples were taken from this solution immediately after incubation, as well as after 8 h of recovery at room temperature. A third sample was taken from a solution incubated at room temperature as control. While the solution incubated at room temperature contained a dense network of fibrillar assemblies with characteristic morphology (Figure 6a), the solution incubated at 37 °C contained fewer assemblies from which fine fibrillar protrusions were extending (Figure 6b). This morphological transition does not seem to be reversible as the characteristic morphology was not fully retained following recovery in room temperature (Figure 6c). Our impression that a lower abundance of assemblies is observed by TEM following incubation at 37 °C was confirmed by a turbidity assay. We incubated 6 mM peptide solutions at 25 °C overnight then separately measured their turbidity over time at 25 °C, 37 °C or 50 °C. While measurements at 25 °C resulted in a slight increase in turbidity, measurements at 37 °C or 50 °C showed a decrease in turbidity over approximately 40 min until a plateau was reached (Figure S3); the measured turbidity did not change further until the end of the experimental timeframe. This result confirms that the assemblies are impaired at elevated temperatures. In accordance with the CD results, it can therefore be concluded that a conformational change occurs at elevated temperatures which leads to an ultrastructural impairment of the fibrillar assemblies.

### 2.3. DOPA-Containing Peptide Assemblies Exhibit Functionality and Reduce Ionic Silver

**Figure 7:**
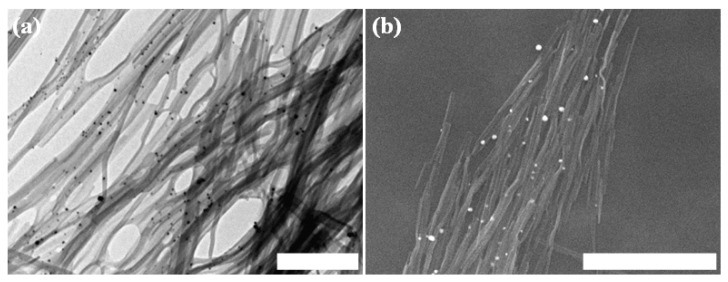
Silver reduction by the Asp-DOPA-Asn-Lys-DOPA fibrillar assemblies. (**a**) TEM micrograph, negative staining was not applied. Scale bar represents 500 nm; (**b**) respective SEM micrograph, metallic coating by sputtering was not applied. Scale bar represents 2 µm.

Silver nanoparticles (AgNP) are a multifunctional material with applications in optics [62], in printed electronics [63] and as antimicrobials [64]. The reduction of ionic silver to AgNP by short peptides is a well-known phenomenon. Previous studies have demonstrated that catechol-containing compounds can reduce ionic silver to nanoparticles or deposited clusters [35,43,65,66]. We therefore examined the ability of Asp-DOPA-Asn-Lys-DOPA assemblies to reduce ionic silver. Peptide solution was produced by means of repeated pelleting in water; this was performed in order to remove peptide monomers. AgNO_3_ solution was then used for resuspention of the pellet and the solution was incubated then re-pelleted and resuspended in water. TEM examination of the resultant solution revealed significant deposition of silver on the fibrillar assemblies which appeared as dark nanometric clusters (Figure 7a), while this was not observed in a control solution to which AgNO_3_ was not added. Furthermore, the clusters seemed to have selectively deposited on the assemblies compared to the background. Similar results were obtained when the sample was examined by SEM, with the clusters appearing in white (Figure 7b). Energy-dispersive X-ray analysis (EDX) of the white clusters confirmed the presence of silver (Figure S4). These results show that Asp-DOPA-Asn-Lys-DOPA assemblies possess the ability to reduce ionic silver while retaining their ultrastructure in solution.

## 3. Experimental Section

Asp-DOPA-Asn-Lys-DOPA pentapeptide was synthesized by Peptron Inc. (Daejeon, Korea) and the Asp-Phe-Asn-Lys-Phe pentapeptide was synthesized by Peptide 2.0 Inc. (Chantilly, VA, USA). To induce the formation of fibrillar assemblies, lyophilized peptide was dissolved in ultra-pure water to concentrations of 100 µM to 15 mM by vortexing followed by bath-sonication for 10 min. To avoid pre-aggregation, fresh stock solutions were prepared for each experiment.

Transmission electron microscopy (TEM) was performed with 10 μL samples. Freshly prepared samples were applied to 400-mesh copper grids covered by carbon-stabilized Formvar film (Electron Microscopy Science, Fort Washington, PA, USA) and allowed to adsorb for 2 min before excess fluid was blotted off. For samples that were negatively stained, 10 μL of 2% uranyl acetate were then deposited on the grid and allowed to adsorb for 2 min before excess fluid was blotted off. TEM micrographs were recorded using a JEOL 1200EX electron microscope (JEOL, Tokyo, Japan) operating at 80 kV.

Scanning electron microscopy (SEM) analysis was performed by using samples deposited on TEM grids as described above without applying negative staining and without applying any metallic coating to the sample. Grids were placed on the microscope metal stand and images were taken using Quanta 200 Field Emission Gun (FEG) Environmental SEM microscope (FEI, Eindhoven, The Netherlands) operating at 20 kV. For further analysis by EDX, Oxford INCA (Oxford Instrument America Inc., Concord, MA, USA) was used.

Congo Red staining was performed with 10 μL samples of 6 mM peptide solution. The samples were air-dried on glass microscope slides and staining was performed by the addition of 10 μL solution of 80% ethanol saturated with Congo Red and NaCl. Birefringence was determined using an Olympus SZX-12 Stereoscope (Olympus, Hamburg, Germany) equipped with a polarizing stage. Fluorescence visualization was performed using Nikon Eclipse 80i epifluorescent microscope (Nikon, Kanagawa, Japan) equipped with a Y-2E/C filter set (excitation 560/20 nm, emission 630/30 nm). Thioflavin-T (ThT) staining was performed by adding fresh 4 mM ThT solution to an equal volume of 6 mM peptide solution which was incubated for 3 h prior to the addition of ThT. The resultant solution was incubated for 3 h in the dark and 10 µL samples were imaged using LSM 510 Meta confocal microscope (Carl Zeiss, Oberkochen, Germany) at 458 nm excitation and 485 nm emission.

Fourier transform infrared (FTIR) spectroscopy was performed with 30 μL samples of 6 mM peptide that was dissolved in deuterium oxide (99.8%, Sigma-Aldrich, Rehovot, Israel) and incubated overnight. The samples were deposited onto disposable polyethylene IR sample cards (Sigma-Aldrich, Israel) which were then allowed to dry under vacuum. Transmission infrared spectra were collected using Nexus 470 FTIR spectrometer (Nicolet, Offenbach, Germany) with a deuterated triglycine sulfate (DTGS) detector. Measurements were made using the atmospheric suppression mode, by averaging 64 scans in 2 cm^−1^ resolution. The amide I’ region was deconvoluted by subtracting a baseline of deuterium oxide that was deposited on a polyethylene sample card and dried under vacuum. Subtraction was performed using the OMNIC software (Nicolet, Offenbach, Germany). Smoothing, second derivative calculation and curve-fitting were then performed using the Peakfit software version 4.12 (SYSTAT, Richmond, CA, USA).

Circular dichroism (CD) spectroscopy was performed by diluting fresh 6 mM peptide solutions in ultra-pure water to a concentration of 3 mM. CD spectra were collected with a Chirascan spectrometer (Applied Photophysics, Leatherhead, UK) fitted with a Peltier temperature controller set to 25 °C, using a capped rectangular quartz cuvette with an optical path length of 0.1 cm. Absorbance was kept at the linear range of the instrument during all measurements. Data acquisition was performed in steps of 1 nm at a wavelength range from 190 to 260 nm with a spectral bandwidth of 1.0 nm and an averaging time of 3 s. The spectrum of each sample was collected three times and a control spectrum of ultra-pure water was collected twice. Spectra were corrected in baseline with ultra-pure water as the blank. Data processing was done using Pro-Data Viewer software (Applied Photophysics, Leatherhead, UK); processing and normalization to mean residue ellipticity (MRE) was performed as described previously [67]. To verify the assayed solution contained characteristic assemblies, a 10 μL sample of the cuvette content was examined by TEM as described above.

Temperature-dependent CD was performed using freshly made 6 mM peptide solution diluted to a concentration of 0.15 mM. CD spectra were collected as described above except that the spectra were obtained throughout temperature variation done in a stepwise fashion up and then down. The investigated temperatures ranged over 25–90 °C (in the following steps: 18 °C, 25 °C, 37 °C, 50 °C, 70 °C, 90 °C, 70 °C, 50 °C, 37 °C, 25 °C, 18 °C). At each temperature point, the sample was allowed to equilibrate for 10 min while the temperature was monitored by a thermocouple in the cuvette holder block. At the end of the measurements, the cuvette content was sampled for TEM analysis as described above. As control, TEM samples were taken from an aliquot of the same solution which was not subjected to temperature variations.

Turbidity assay was performed for 6 mM Asp-DOPA-Asn-Lys-DOPA solutions that were incubated overnight at 25 °C. Optical density at 350 nm was measured using a Biotek Synergy HT plate reader (Biotek, Winooski, VT, USA) over a time period of 4 h at 25 °C, 37 °C or 50 °C. For each temperature point, an individual experiment was performed by pipetting 100 µL peptide solution in triplicates into a 96-well UV-Star UV transparent plate (Greiner BioOne, Frickenhausen, Germany). The results of each experiment were averaged and normalized separately.

Silver deposition was done by preparing 6 or 15 mM peptide solutions and removing residual peptide monomers by centrifugation at 12,000 RPM for 15 min, discarding the supernatant and resuspention in ultra-pure water. This procedure was repeated once more. Samples for electron microscopy were taken as control and the solution was centrifuged at 12,000 RPM for 15 min and resuspended in an aqueous solution of 2.17 mM AgNO_3_ for 15 min. Finally, the solution was centrifuged at 9000 RPM for 10 min and resuspended in water. Samples for electron microscopy were taken again. SEM and EDX analyses were performed as described above.

## 4. Conclusions

We report that a DOPA-incorporated pentapeptide inspired by a minimal amyloid recognition module can self-assemble into an amyloid-like supramolecular polymer of fibrillar nature in water. The assemblies formed were investigated by electron microscopy, amyloidophilic dyes and spectroscopic methods. The investigation revealed that the supramolecular polymer formed is endowed with characteristics of both amyloids and DOPA-containing polypeptides. Furthermore, the ability to reduce ionic silver while maintaining the ultrastructural integrity has demonstrated the applicative potential of this novel nanobiomaterial.

## Supplementary Materials

Supplementary File 1

## References

[1] McGaughey G.B., Gagné M., Rappé A.K. (1998). π-stacking interactions alive and well in proteins. J. Biol. Chem..

[2] Zhang S. (2002). Emerging biological materials through molecular self-assembly. Biotechnol. Adv..

[3] Dobson C.M. (1999). Protein misfolding, evolution and disease. Trends. Biochem. Sci..

[4] Harper J.D., Lansbury P.T. (1997). Models of amyloid seeding in Alzheimer’s disease and scrapie: Mechanistic truths and physiological consequences of the time-dependent solubility of amyloid proteins. Annu. Rev. Biochem..

[5] Serpell L.C. (2000). Alzheimer’s amyloid fibrils: Structure and assembly. BBA-Mol. Basis Dis..

[6] Geddes A.J., Parker K.D., Atkins E.D.T., Beighton E. (1968). “Cross-β” conformation in proteins. J. Mol. Biol..

[7] Nelson R., Sawaya M.R., Balbirnie M., Madsen A.Ø., Riekel C., Grothe R., Eisenberg D. (2005). Structure of the cross-β spine of amyloid-like fibrils. Nature.

[8] Gazit E. (2005). Mechanisms of amyloid fibril self-assembly and inhibition. FEBS J..

[9] Balbach J.J., Ishii Y., Antzutkin O.N., Leapman R.D., Rizzo N.W., Dyda F., Reed J., Tycko R. (2000). Amyloid fibril formation by Aβ_16–22_, a seven-residue fragment of the Alzheimer’s β-amyloid peptide, and structural characterization by solid state NMR. Biochemistry.

[10] Madine J., Doig A.J., Middleton D.A. (2008). Design of an n-methylated peptide inhibitor of α-synuclein aggregation guided by solid-state NMR. J. Am. Chem. Soc..

[11] Mazor Y., Gilead S., Benhar I., Gazit E. (2002). Identification and characterization of a novel molecular-recognition and self-assembly domain within the islet amyloid polypeptide. J. Mol. Biol..

[12] Reches M., Gazit E. (2004). Amyloidogenic hexapeptide fragment of medin: Homology to functional islet amyloid polypeptide fragments. Amyloid.

[13] Sawaya M.R., Sambashivan S., Nelson R., Ivanova M.I., Sievers S.A., Apostol M.I., Thompson M.J., Balbirnie M., Wiltzius J.J., McFarlane H.T. (2007). Atomic structures of amyloid cross-β spines reveal varied steric zippers. Nature.

[14] MacIntyre I. (1967). Calcitonin: A general review. Calcif. Tissue Int..

[15] Sletten K., Westermark P., Natvig J.B. (1976). Characterization of amyloid fibril proteins from medullary carcinoma of the thyroid. J. Exp. Med..

[16] Kedar I., Ravid M., Sohar E. (1976). *In vitro* synthesis of “amyloid” fibrils from insulin, calcitonin and parathormone. Isr. J. Med. Sci..

[17] Kanaori K., Nosaka A.Y. (1995). Study of human calcitonin fibrillation by proton nuclear magnetic resonance spectroscopy. Biochemistry.

[18] Kamihira M., Naito A., Tuzi S., Nosaka A.Y., Saito H. (2000). Conformational transitions and fibrillation mechanism of human calcitonin as studied by high-resolution solid-state ^13^C NMR. Protein Sci..

[19] Reches M., Porat Y., Gazit E. (2002). Amyloid fibril formation by pentapeptide and tetrapeptide fragments of human calcitonin. J. Biol. Chem..

[20] Gavin Tsai H.-H., Zanuy D., Haspel N., Gunasekaran K., Ma B., Tsai C.J., Nussinov R. (2004). The stability and dynamics of the human calcitonin amyloid peptide DFNKF. Biophys. J..

[21] Naito A., Kamihira M., Inoue R., Saitô H. (2004). Structural diversity of amyloid fibril formed in human calcitonin as revealed by site-directed ^13^C solid-state NMR spectroscopy. Magn. Reson. Chem..

[22] Haspel N., Zanuy D., Ma B., Wolfson H., Nussinov R. (2005). A comparative study of amyloid fibril formation by residues 15–19 of the human calcitonin hormone: A single β-sheet model with a small hydrophobic core. J. Mol. Biol..

[23] Pak Y., Shin J., Jang S. (2009). Computational study of human calcitonin (hCT) oligomer. Bull. Korean Chem. Soc..

[24] Cherny I., Gazit E. (2008). Amyloids: Not only pathological agents but also ordered nanomaterials. Angew. Chem. Int. Ed..

[25] Pilkington S.M., Roberts S.J., Meade S.J., Gerrard J.A. (2010). Amyloid fibrils as a nanoscaffold for enzyme immobilization. Biotechnol. Prog..

[26] Hauser C.A.E., Maurer-Stroh S., Martins I.C. (2014). Amyloid-based nanosensors and nanodevices. Chem. Soc. Rev..

[27] Reches M., Gazit E. (2003). Casting metal nanowires within discrete self-assembled peptide nanotubes. Science.

[28] Scheibel T., Parthasarathy R., Sawicki G., Lin X.-M., Jaeger H., Lindquist S.L. (2003). Conducting nanowires built by controlled self-assembly of amyloid fibers and selective metal deposition. Proc. Natl. Acad. Sci. USA.

[29] Orbach R., Adler-Abramovich L., Zigerson S., Mironi-Harpaz I., Seliktar D., Gazit E. (2009). Self-assembled Fmoc-peptides as a platform for the formation of nanostructures and hydrogels. Biomacromolecules.

[30] Scheibel T. (2005). Protein fibers as performance proteins: New technologies and applications. Curr. Opin. Biotechnol..

[31] Fichman G., Gazit E. (2014). Self-assembly of short peptides to from hydrogels: Design of building blocks, physical propertiesand technological applications. Acta Biomater..

[32] Reithofer M.R., Chan K.-H., Lakshmanan A., Lam D.H., Mishra A., Gopalan B., Joshi M., Wang S., Hauser C.A.E. (2014). Ligation of anti-cancer drugs to self-assembling ultrashort peptides by click chemistry for localized therapy. Chem. Sci..

[33] Wiegemann M. (2005). Adhesion in blue mussels (mytilus edulis) and barnacles (genus balanus): Mechanisms and technical applications. Aquat. Sci..

[34] Yu M., Hwang J., Deming T.J. (1999). Role of L-3,4-dihydroxyphenylalanine in mussel adhesive proteins. J. Am. Chem. Soc..

[35] Lee H., Dellatore S.M., Miller W.M., Messersmith P.B. (2007). Mussel-inspired surface chemistry for multifunctional coatings. Science.

[36] Xu C., Xu K., Gu H., Zheng R., Liu H., Zhang X., Guo Z., Xu B. (2004). Dopamine as a robust anchor to immobilize functional molecules on the iron oxide shell of magnetic nanoparticles. J. Am. Chem. Soc..

[37] Dalsin J.L., Hu B.-H., Lee B.P., Messersmith P.B. (2003). Mussel adhesive protein mimetic polymers for the preparation of nonfouling surfaces. J. Am. Chem. Soc..

[38] Black K.C.L., Liu Z., Messersmith P.B. (2011). Catechol redox induced formation of metal core-polymer shell nanoparticles. Chem. Mater..

[39] Charlot A., Sciannaméa V., Lenoir S., Faure E., Jérôme R., Jérôme C., van de Weerdt C., Martial J., Archambeau C., Willet N. (2009). All-in-one strategy for the fabrication of antimicrobial biomimetic films on stainless steel. J. Mater. Chem..

[40] Fullenkamp D.E., Rivera J.G., Gong Y.-K., Lau K.H.A., He L., Varshney R., Messersmith P.B. (2012). Mussel-inspired silver-releasing antibacterial hydrogels. Biomaterials.

[41] Saha A., Bolisetty S., Handschin S., Mezzenga R. (2013). Self-assembly and fibrillization of a Fmoc-functionalized polyphenolic amino acid. Soft Matter.

[42] Ceylan H., Urel M., Erkal T.S., Tekinay A.B., Dana A., Guler M.O. (2013). Mussel inspired dynamic cross-linking of self-healing peptide nanofiber network. Adv. Funct. Mater..

[43] Fichman G., Adler-Abramovich L., Manohar S., Mironi-Harpaz I., Guterman T., Seliktar D., Messersmith P.B., Gazit E. (2014). Seamless metallic coating and surface adhesion of self-assembled bio-inspired nanostructures based on di-(3,4-dihydroxy-L-phenylalanine) peptide motif. ACS Nano.

[44] Howie A.J., Brewer D.B. (2009). Optical properties of amyloid stained by Congo red: History and mechanisms. Micron.

[45] Liang G., Xu K., Li L., Wang L., Kuang Y., Yang Z., Xu B. (2007). Using Congo red to report intracellular hydrogelation resulted from self-assembly of small molecules. Chem. Commun..

[46] Hamill A.C., Wang S.-C., Lee C.T. (2007). Solution structure of an amyloid-forming protein during photoinitiated hexamer-dodecamer transitions revealed through small-angle neutron scattering. Biochemistry.

[47] Naiki H., Higuchi K., Hosokawa M., Takeda T. (1989). Fluorometric determination of amyloid fibrils *in vitro* using the fluorescent dye, thioflavine T. Anal. Biochem..

[48] Levine H. (1993). Thioflavine T teraction with synthetic Alzheimer’s disease β-amyloid peptides: Detection of amyloid aggregation in solution. Protein Sci..

[49] Biancalana M., Koide S. (2010). Molecular mechanism of thioflavin-T binding to amyloid fibrils. Biochim. Biophys. Acta.

[50] Shtainfeld A., Sheynis T., Jelinek R. (2010). Specific mutations alter fibrillation kinetics, fiber morphologies, and membrane interactions of pentapeptides derived from human calcitonin. Biochemistry.

[51] Byler D.M., Susi H. (1986). Examination of the secondary structure of proteins by deconvolved FTIR spectra. Biopolymers.

[52] Kong J., Yu S. (2007). Fourier transform infrared spectroscopic analysis of protein secondary structures. Acta Biochim. Biophy. Sin..

[53] Surewicz W.K., Mantsch H.H. (1988). New insight into protein secondary structure from resolution-enhanced infrared spectra. Biochim. Biophys. Acta.

[54] Gilmanshin R., Williams S., Callender R.H., Woodruff W.H., Dyer R.B. (1997). Fast events in protein folding: Relaxation dynamics and structure of the I form of apomyoglobin. Biochemistry.

[55] Manas E.S., Getahun Z., Wright W.W., DeGrado W.F., Vanderkooi J.M. (2000). Infrared spectra of amide groups in α-helical proteins: Evidence for hydrogen bonding between helices and water. J. Am. Chem. Soc..

[56] Yamamoto H., Hayakawa T. (1983). Conformational studies of sequential polypeptides containing L-β-(3,4-dihydroxyphenyl)-α-alanine (dopa) and L-lysine. Macromolecules.

[57] Dutt A., Dutta A., Mondal R., Spencer E.C., Howard J.A.K., Pramanik A. (2007). Studies of β-turn opening with model peptides containing non-coded α-amino isobutyric acid. Tetrahedron.

[58] Kar S., Dutta A., Drew M.G.B., Koley P., Pramanik A. (2009). Design of supramolecular β-sheet forming β-turns containing aromatic rings and non-coded α-aminoisobutyric acid and the formation of flat fibrillar structures through self-assembly. Supramol. Chem..

[59] Maji S.K., Haldar D., Drew M.G.B., Banerjee A., Das A.K., Banerjee A. (2004). Self-assembly of β-turn forming synthetic tripeptides into supramolecular β-sheets and amyloid-like fibrils in the solid state. Tetrahedron.

[60] Lakshmanan A., Cheong D.W., Accardo A., di Fabrizio E., Riekel C., Hauser C.A.E. (2013). Aliphatic peptides show similar self-assembly to amyloid core sequences, challenging the importance of aromatic interactions in amyloidosis. Proc. Natl. Acad. Sci. USA.

[61] Tifany M.L., Krimm S. (1972). Effect of temperature on the circular dichroism spectra of polypeptides in the extended state. Biopolymers.

[62] McFarland A.D., van Duyne R.P. (2003). Single silver nanoparticles as real-time optical sensors with zeptomole sensitivity. Nano Lett..

[63] Li Y., Wu Y., Ong B.S. (2005). Facile synthesis of silver nanoparticles useful for fabrication of high-conductivity elements for printed electronics. J. Am. Chem. Soc..

[64] Rai M., Yadav A., Gade A. (2009). Silver nanoparticles as a new generation of antimicrobials. Biotechnol. Adv..

[65] Liao Y., Wang Y., Feng X., Wang W., Xu F., Zhang L. (2010). Antibacterial surfaces through dopamine functionalization and silver nanoparticle immobilization. Mater. Chem. Phys..

[66] Xu H., Shi X., Ma H., Lv Y., Zhang L., Mao Z. (2011). The preparation and antibacterial effects of DOPA-cotton/AgNPs. Appl. Surf. Sci..

[67] Greenfield N.J. (2007). Using circular dichroism spectra to estimate protein secondary structure. Nat. Protoc..

