# Supplementary material for: The Use of the Calcitonin Minimal Recognition Module for the Design of DOPA-Containing Fibrillar Assemblies
Source: Nanomaterials (Basel). 2014 Aug 20;4(3):726–40. doi: 10.3390/nano4030726 (PMC5304689; doi:10.3390/nano4030726)

Supporting Information

Figure S1. TEM micrograph of the unmodified hCT minimal recognition module, Asp-Phe-Asn-Lys-Phe. The micrograph is of 6 mM peptide in water prepared similarly to Asp-DOPA-Asn-Lys-DOPA. Negative staining was applied. Scale bar represents 100 nm.

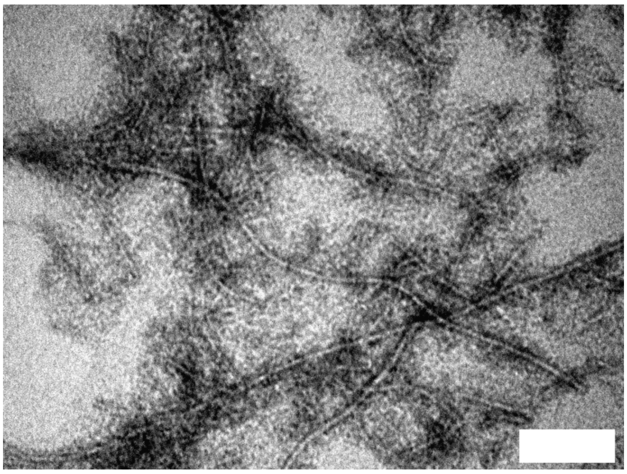

Figure S2. Thioflavin-T (ThT) staining of Asp-DOPA-Asn-Lys-DOPA. 6 mM peptide solution was allowed to assemble for 3 h then incubated for 3 h with an equal volume of 4 mM ThT. Sample was imaged using a confocal laser scanning microscope; **(a)** ThT fluorescence; **(b)** Differential image contrast (DIC); **(c)** Overlay of the previous images.

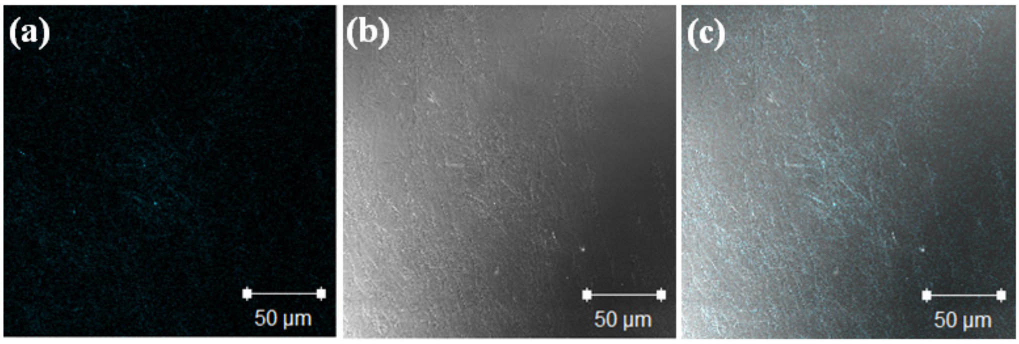

Figure S3. Turbidity assay of 6 mM Asp-DOPA-Asn-Lys-DOPA in water at 25 °C, 37 °C or 50 °C. Optical density was monitored at 350 nm for 4 h; the figure represents the first 90 min of the experiment as the signal did not change afterwards for the higher temperatures.

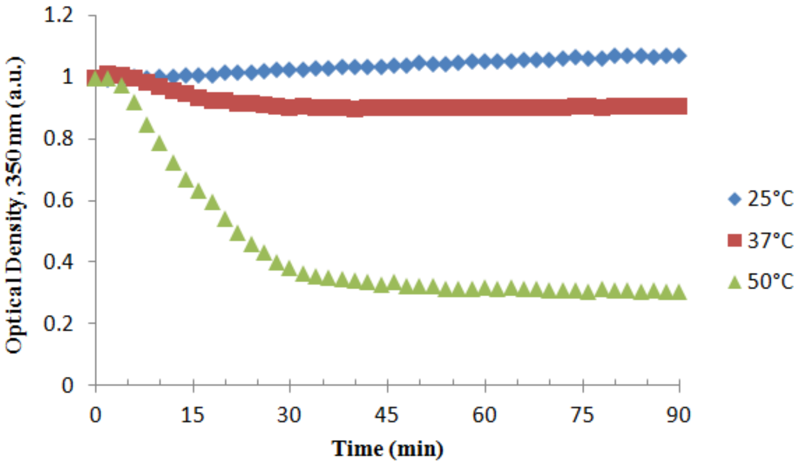

Figure S4. Energy-dispersive X-ray analysis of Asp-DOPA-Asn-Lys-DOPA fibrillar assemblies deposited by silver. The presence of copper is due to the deposition of the sample on a copper TEM grid.

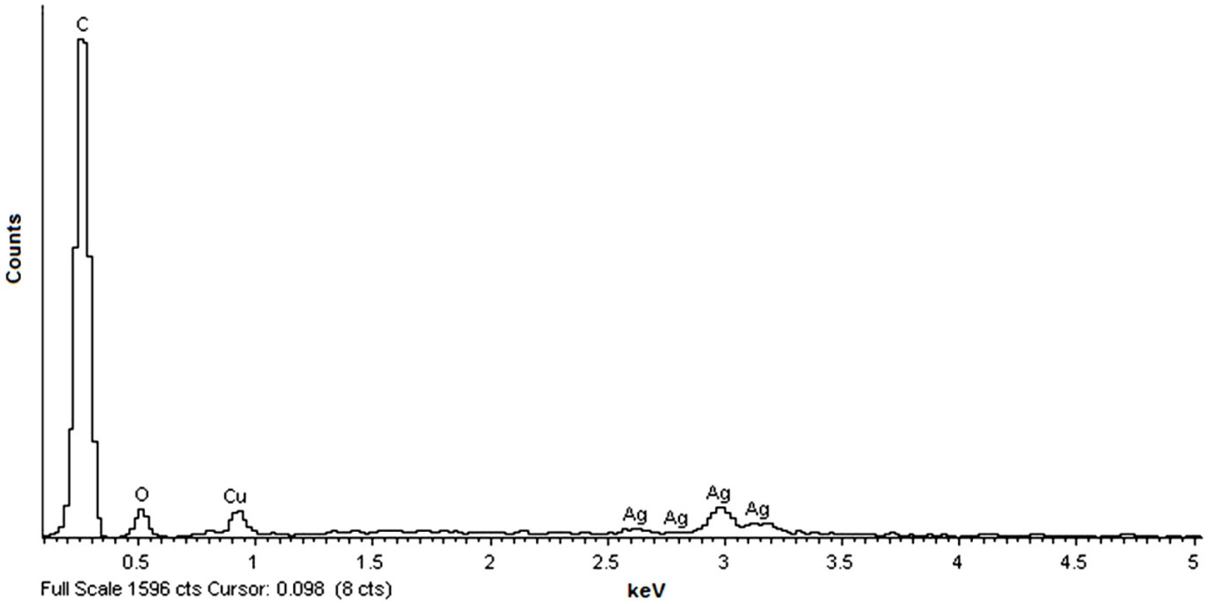

Supplement: Supplementary File 1 [file nanomaterials-04-00726-s001.pdf]
